# Multi-Modality Therapeutics with Potent Anti-Tumor Effects: Photochemical Internalization Enhances Delivery of the Fusion Toxin scFvMEL/rGel

**DOI:** 10.1371/journal.pone.0006691

**Published:** 2009-08-19

**Authors:** Pål K. Selbo, Michael G. Rosenblum, Lawrence H. Cheung, Wendy Zhang, Kristian Berg

**Affiliations:** 1 Department of Radiation Biology, Institute for Cancer Research, Norwegian Radium Hospital, Oslo University Hospital, Oslo, Norway; 2 Immunopharmacology and Targeted Therapy Laboratory, Department of Experimental Therapeutics, M. D. Anderson Cancer Center, Houston, Texas, United States of America; Bauer Research Foundation, United States of America

## Abstract

**Background:**

There is a need for drug delivery systems (DDS) that can enhance cytosolic delivery of anti-cancer drugs trapped in the endo-lysosomal compartments. Exposure of cells to specific photosensitizers followed by light exposure (photochemical internalization, PCI) results in transfer of agents from the endocytic compartment into the cytosol.

**Methodology and Principal Findings:**

The recombinant single-chain fusion construct scFvMEL/rGel is composed of an antibody targeting the progenitor marker HMW-MAA/NG2/MGP/gp240 and the highly effective toxin gelonin (rGel). Here we demonstrate enhanced tumor cell selectivity, cytosolic delivery and anti-tumor activity by applying PCI of scFvMEL/rGel. PCI performed by light activation of cells co-incubated with scFvMEL/rGel and the endo-lysosomal targeting photosensitizers AlPcS_2a_ or TPPS_2a_ resulted in enhanced cytotoxic effects against antigen-positive cell lines, while no differences in cytotoxicity between the scFvMEL/rGel and rGel were observed in antigen-negative cells. Mice bearing well-developed melanoma (A-375) xenografts (50–100 mm^3^) were treated with PCI of scFvMEL/rGel. By 30 days after injection, ∼100% of mice in the control groups had tumors>800 mm^3^. In contrast, by day 40, 50% of mice in the PCI of scFvMEL/rGel combination group had tumors<800 mm^3^ with no increase in tumor size up to 110 days. PCI of scFvMEL/rGel resulted in a synergistic effect (p<0.05) and complete regression (CR) in 33% of tumor-bearing mice (n = 12).

**Conclusions/Significance:**

This is a unique demonstration that a non-invasive multi-modality approach combining a recombinant, targeted therapeutic such as scFvMEL/rGel and PCI act in concert to provide potent *in vivo* efficacy without sacrificing selectivity or enhancing toxicity. The present DDS warrants further evaluation of its clinical potential.

## Introduction

The emergence of membrane impermeable or macromolecular-based biopharmaceuticals with intracellular targets has initiated a need for smart drug delivery systems (DDS). Sequestration and degradation of such anti-cancer therapeutics in endosomes or lysosomes is a major barrier for efficient cancer therapy [1]. To achieve sufficient cytosolic or nuclear concentrations in the target cells, high doses of anti-neoplastic drugs are frequently administered leading to undesired adverse effects. Therefore, current efforts in cancer pharmaceutics include the development of targeted DDS in which the drug of interest is activated only in the malignant tissue and thereby sparing normal tissue.

We have established photochemical internalization (PCI) as a novel DDS for the controlled cytosolic release of macromolecules or chemotherapeutic agents that are sequestered in endo-lysosomal compartments. PCI is based on photodynamic therapy (PDT), a photochemical method generating reactive oxygen species (ROS) after confined light activation of a tumor-accumulating photosensitizer [2]–[4]. The main PDT-induced ROS product is singlet oxygen, which can destroy a number of biomolecules including lipids and proteins of the endo-lysosomal membranes [5], [6]. Hence, PCI provide a unique, controlled, spatio-temporal DDS in which intracellular molecules held in the endosomal/lysosomal compartment are released to the cytosol by the effects of PCI.

The progenitor marker gp240, also known as HMW-MAA/NG2/MPG/MCSP, has emerged as a potential target for cancer therapy due to its pro-survival, growth and invasion signaling in several cancers types that exert broad resistance profiles against chemotherapy [7]–[10]. gp240 is expressed in >80% of human melanomas [11] and 67% of lobular breast carcinomas [12] and has been associated with angiogenesis in brain tumors [13] where it seems to be expressed on hematopoietic stem cell-derived pericytes [14]. In addition, gp240 is recognized as a marker for multipotent early oligodendrocyte progenitors in normal CNS [15], [16] and its increased co-expression with CD44 has been proposed as progressive marker of malignant gliomas [17]. Moreover, gp240 correlates with poor clinical outcome in childhood acute myeloid leukemias [7].

The antibody ZME-018 has been used in numerous studies for imaging [18] and as a carrier for development of gp240-targeted therapeutic approaches. Rosenblum et al. have developed chemical conjugates of full-length ZME-018 and toxins such as rGel, nGel and TNF. In addition, recombinant single-chain antibodies targeting gp240 and have been fused to payloads such as rGel, TNF and granzyme B [19]–[21]. The scFvMEL/rGel fusion construct has been well-characterized and demonstrates impressive and selective cytotoxic effects against tumor cells in culture and against tumor xenograft models.

We proposed that PCI may augment the biological activity of many agents including tumor cell targeted therapeutics and fusion constructs. This study describes the first report of the use of PCI as a modality to enhance the delivery of a tumor-targeting drug *in vivo*. PCI was found to induce cytosolic release of the internalized fusion construct and strongly potentiated the anti-tumor effect of the recombinant fusion toxin scFvMEL/rGel against tumor xenografts. There were no adverse side effects observed in nude mice and these studies clearly demonstrate that using PCI as a modality may greatly improve the efficacy of cell-targeted therapeutic agents.

## Results

### Cytosolic delivery of photosensitizer and scFvMEL/rGel upon light exposure

**Figure 1:**
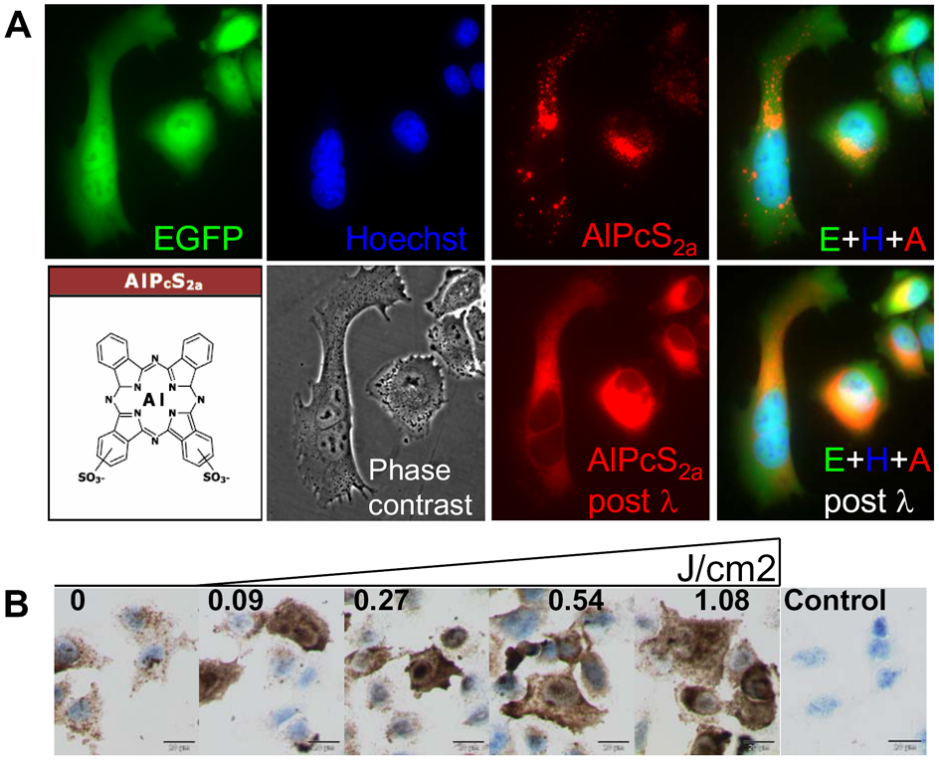
Photochemical-mediated endo-lysosomal release and cytosolic delivery of photosensitizer and scFvMEL/rGel. A375-GFP cells were incubated for 18 h with 5 µg/ml AlPcS_2a_, washed twice and chased in drug-free medium for 4 h. 30 min prior to microscopy, cells were incubated with 5 µg/ml Hoechst 33342. A, Fluorescence of EGFP, Hoechst 33342 and AlPcS_2a_ and merged (E+H+A) in vital cells prior to (upper panels) and 1 min post 10 sec of microscopy red light exposure (590–650 nm) with respectively merged micrographs and corresponding phase contrast (mid panels). Structure formula of AlPcS_2a_ in left lower panel. B, A-375 cells were co-incubated for 18 h with 80 nM scFvMEL/rGel and 5 µg/ml AlPcS_2a_, washed twice and chased in drug-free medium for 4 h prior to light exposure and further incubated at 37°C for 30 min prior to ICC detection of rGel. Right panel, negative controls included rabbit IgG isotype.

Cytosolic release of AlPcS_2a_ post light exposure (PDT) was evaluated by epi-fluorescence microscopy of A375-GFP cells, while recombinant gelonin (rGel) fused to the scFv-fragment (scFvMEL/rGel), was detected by ICC. Granular red fluorescence (>660 nm) of AlPcS_2a_ characteristic for endocytic vesicles was detected in the A375-GFP cells (Figure 1A). Red light exposure of the AlPcS_2a_-treated A375-GFP cells resulted in an immediate relocalization of the photosensitizer throughout the cytosol of the cells. No detectable overlap of fluorescence from the photosensitizer (red) and Hoechst 33342 (blue) was detected, indicating extranuclear localization of AlPcS_2a_.

Prior to PCI of scFvMEL/rGel, a distinct granular extranuclear localization of the rGel was detected by ICC of A-375 cells (Figure 1B). Thirty minutes after PCI of scFvMEL/rGel, a significant light-dose-dependent redistribution of rGel to the whole cell was observed, demonstrating release of scFvMEL/rGel into the cytoplasm and nucleoplasm. Positive and negative controls to verify specific detection of rGel, as described in Materials and Methods, gave satisfactory results.

### Cytotoxicity of PCI of scFvMEL/rGel in A-375 melanoma cells

The measurements of cytotoxicity of PCI were mostly performed by the MTT assay, however confirmed by either clonal cell survival or by the assessment of GFP-signals since leakage of GFP has been reported to correlate with cell death [22], [23].

**Figure 2:**
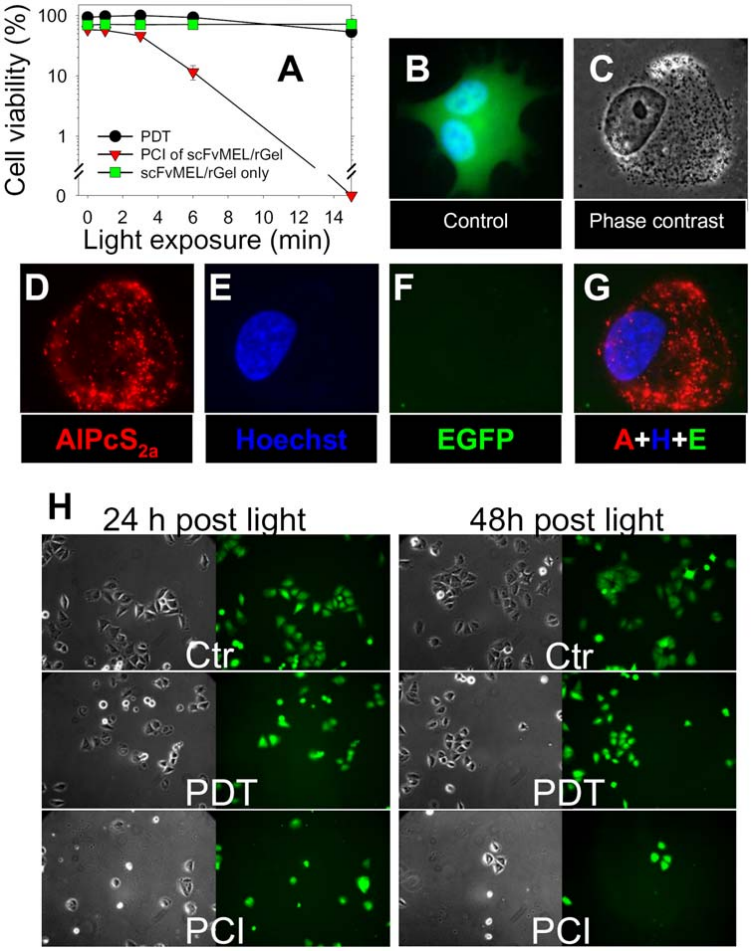
Enhancement of cytotoxicity and reduction of EGFP signals by PCI of scFvMEL/rGel in A375-GFP melanoma cells. A, Viability of A375-GFP cells subjected to PCI of scFvMEL/rGel (red triangles), PDT (black dots) or scFvMEL/rGel only (green squares). Cells were incubated with 5 µg/ml AlPcS_2a_ with or without 100 nM scFvMEL/rGel for 18 h, followed by 3 times wash and 4 h chase in drug-free medium prior to exposure to increasing light exposure of cells (15 minutes = 1.35 J/cm^2^). Cytotoxicity was assessed by the MTT assay 48 h post light exposure. Bars, SD. B, Fluorescence of EGFP and Hoechst 33342 in an untreated A375-GFP cell. C, Phase contrast of a cell treated with PCI of scFvMEL/rGel (LD90, 0.54 J/cm^2^) with corresponding fluorescence of AlPcS_2a_ (D), Hoechst 33342 (E), EGFP (F) and a merge of D,E and F (G) 48 h post light exposure. H, A375-GFP cells 24 and 48 hours post either PDT (LD50, 1.35 J/cm^2^) or PCI of scFvMEL/rGel (LD90, 0.54 J/cm^2^) as compared to untreated control cells (Ctr).

The photochemically induced relocalization of rGel (Figure 1B) is in accordance with the enhanced cytotoxicity observed when the photochemical treatment was combined with scFvMEL/rGel treatment in A375-GFP (Figure 2A). PCI performed by combining subtoxic photochemical and scFvMEL/rGel treatment doses resulted in a substantial reduced A375-GFP cell viability as measured by the MTT cell viability assay 48 hrs after light exposure. Similar results were obtained in the maternal A-375 cell line after PCI of scFvMEL/rGel using either TPPS_2a_ (Figure S1) or AlPcS_2a_ (data not shown) as photosensitizers. No differences in toxicity were observed between PCI of non-conjugated nGel and rGel in the A-375 cells and the cytotoxic effects were substantially lower than after PCI of scFvMEL/rGel. Exposure to light in the absence of an externally added photosensitizer did not induce any decrease in cell viability in cells treated with scFvMEL/rGel in both A375-GFP (Figure 2A) and A-375 cells (data not shown).

The photochemical treatment (i.e. PDT) is known to induce a rapid cell inactivation causing cell death within 24 hrs after light exposure [24]. However, in contrast to the MTT cell viability assay performed 48 hrs after light exposure, 24 hrs after the light exposure only a modest attenuation of cell viability was observed by scFvMEL/rGel or rGel in photochemically treated cells as compared to PDT (Figure S2). Similarly, the GFP-signal was lost between 24 and 48 hrs after PCI in A375-GFP cells (Figure 2B–H, Figure S3). Thus, the MTT viability assay correlated with the loss of GFP in the A375-GFP cells. No increase in cell cytotoxicity was observed by postponing the MTT analysis from 48 to 72 hours after light exposure (data not shown). Increased cytotoxic activity measured 48 hours as compared to 24 hours after light exposure indicates that the enzymatic activity of rGel, either as native toxin or as fusion toxin, is a relative slow catalytic process in the cells. Clonal cell survival data analysis confirmed the synergistic effect of PCI of scFvMEL/rGel in the A-375 cells (data not shown).

Previously, we demonstrated, *in vitro* and *in vivo*, that the photochemical delivery of nGel may be performed by administrating nGel shortly after the photochemical treatment [25], [26]. However, the photochemical enhancement of scFvMEL/rGel toxicity was substantially lower when the immunotoxin was delivered directly after the light exposure of the A-375 cells (Figure S4). This is assumed to be due to a photochemical damage to the gp-240 receptor in accordance with the observed photochemical inactivation of EGFR [27], [28].

### Cytotoxicity after PCI of scFvMEL/rGel in different cancer cell lines correlates with the expression of the progenitor marker HMW-MAA/NG2/MPG/gp-240

**Figure 3:**
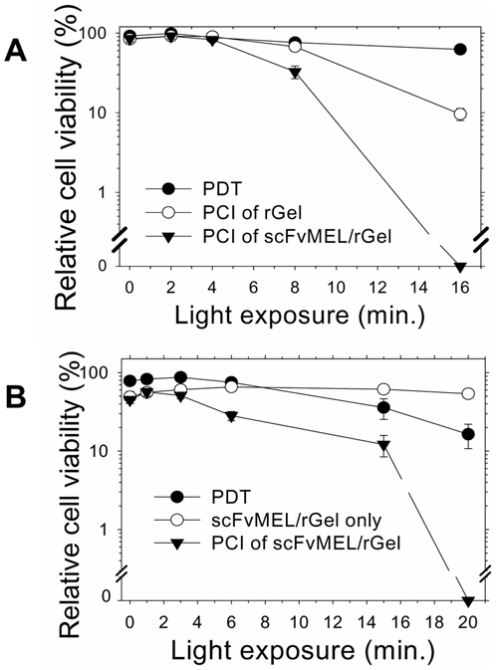
PCI of scFvMEL/rGel in MA11 breast carcinoma cells (A) and U87 malignant glioma cells (B). The cells were incubated with 5.0 µg/ml AlPcS_2a_+/−16.5 nM scFvMEL/rGel or 16.5 nM rGel for 18 hours and then washed twice and chased in drug-free medium 4 h prior to red light exposure. MTT assay was performed 48 hours post light. Experiments with triplicates were reproduced at least twice. Bars, SE.

To demonstrate a general applicability of the PCI-based DDS PCI of scFvMEL/rGel were evaluated in two other cell lines of different cancer origins: Lobular breast carcinoma (MA11) and malignant glioblastoma (U87MG) from brain cancer, the latter shown to express NG2 [9]. Expression of the target receptor on MA11 cells was confirmed by detecting receptor-binding of scFvMEL/rGel by using a primary rabbit antibody against gelonin and an Alexa488 labeled secondary goat anti rabbit antibody, while control experiment using incubation of the primary and the secondary antibody alone resulted in no fluorescing signals (Figure S5). PCI in these cell lines resulted in a strongly enhanced light-dose and toxin-dose dependent cytotoxicity compared to either PDT alone or fusion toxin treatment alone (Figure 3). No dark toxicity of PS alone or in combination with the fusion toxin was observed in both cell lines (Figure 3A).

**Figure 4:**
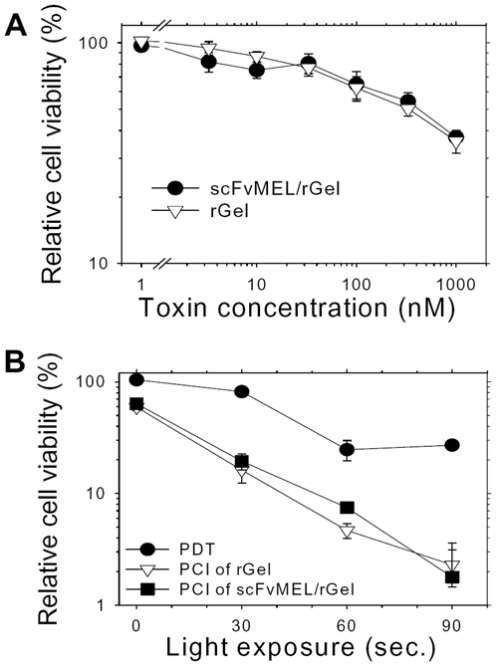
Control experiments in gp240-negative bladder cancer cell line T24. A, Cells were treated with either rGel or scFvMEL/rGel for 18 h with increasing toxin concentrations. B, AlPcS_2a_-PCI of scFvMEL/rGel or rGel in T24 cells. The cells were treated and assessed as described in figure 3. Bars, SE.

As control for gp240-targeting, we compared the cytotoxicity of scFvMEL/rGel with rGel in the gp240 negative T24 bladder carcinoma cell line. No differences in cytotoxicity were observed between the toxin and the immunotoxin treatment in the absence of photochemical treatment (Figure 4A). Accordingly, no enhancement of cytotoxicity was observed after PCI of scFvMEL/rGel as compared to PCI of rGel in the gp-240-negative T24 cells (Figure 4B).

### PCI-enhanced tumor-targeting and anti-tumor activity of scFvMEL/rGel without apparent adverse effects

Subcutaneous A-375 xenografts were used to evaluate the therapeutic effect of PCI of scFvMEL/rGel. PCI was performed by single, systemic administration of AlPcS_2a_ and scFvMEL/rGel prior to light exposure confined to the s.c. tumor as compared to multiple injections which is standard for immunotoxin treatments, including previous work with scFvMEL/rGel [19].

**Figure 5:**
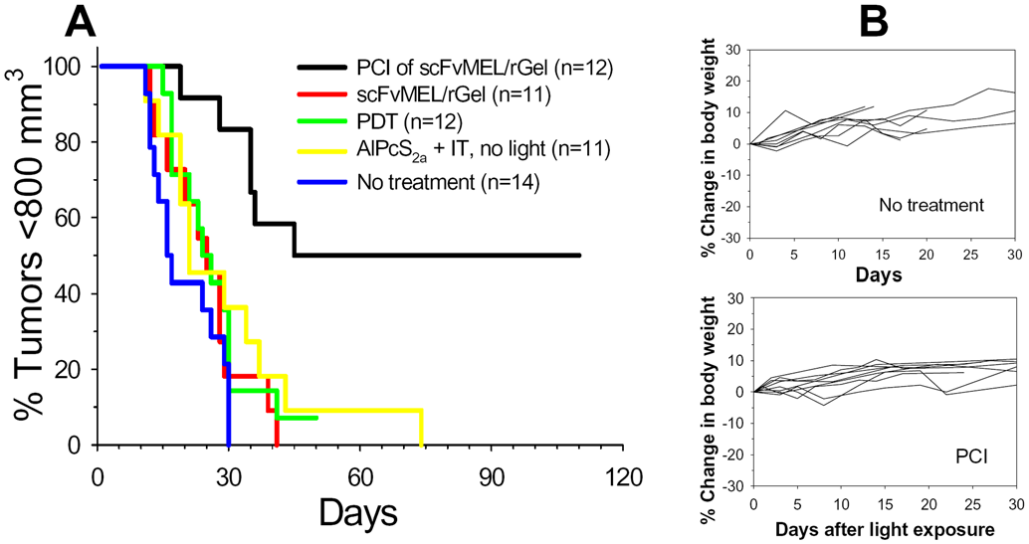
PCI of scFvMEL/rGel in A-375 xenografts. A, Kaplan Meier plot of treatment groups as indicated in the figure. Nude mice with s.c. growing A-375 (50–100 mm3) tumors were given AlPcS_2a_ (5 mg/kg i.p.) with or without scFvMEL/rGel (2 mg/kg i.v) administered 48 and 24 hours prior to laser light exposure (day 0), respectively. The light dose was 20 J/cm^2^ with an irradiance of 100 mW/cm^2^. n, number of animals in each group. B, Weights of the mice treated with PCI of scFvMEL/rGel as compared with non-treated animals. Range of weights at treatment start were 22–29 g. Weight data for the other groups are shown in Figure S6.

The therapeutic potential of PCI of scFvMEL/rGel on A375 xenograft tumors was assessed over time by measuring the effect on tumor growth. The tumor response to the various treatments is presented in a Kaplan-Meier plot in Figure 5. scFvMEL/rGel was found to delay the time for the A-375 xenograft to reach 800 mm3 by 5 days (not significant, Tables 1 and 2). There was a slightly longer delay (10 days), but not significant, in tumor growth of tumors treated with both scFvMEL/rGel and AlPcS_2a_ (Tables 1 and 2). Previous studies on several tumor models have shown that AlPcS_2a_ in the absence of light treatment has no effect on tumor growth [3], [26], [29], [30]. However, the animals are treated and tumors measured under subdued light and it cannot be excluded that the light exposure of the animals during these procedures is sufficient to induce a moderate growth delay in the presence of systemically delivered scFvMEL/rGel. The PDT dose used in this study delayed the tumor growth by approximately 7 days, not significantly different from the growth of the untreated tumors and the other treatments except for the PCI treatment (Tables 1 and 2). The treatment with PCI of scFvMEL/rGel was substantially more effective than all other treatments, inducing a significantly longer growth delay (p≤0,004, Table 2) with 50% of the tumors not reaching the endpoint of 800 mm3 (Figure 5). The PCI of scFvMEL/rGel treatment was found to act in a synergistic manner (p<0.05) as compared to the expected sum of the individual treatments scFvMEL/rGel and PDT with R = 1.15, indicating that the tumor growth delay by PCI of scFvMEL/rGel was more that twice as long as expected by an additive effect. A complete regression (CR) of the A-375 tumors was obtained in 33% of the mice treated with PCI of scFvMEL/rGel.

**Table 1:** Mean time for A-375 tumors to reach the endpoint of 800 mm^3^.

Groups	Treatment	No. of animals	Mean time to reach 800 mm	SE
**1**	**untreated**	**14**	20,0	2,1
**2**	**scFvMEL/rGel**	**11**	24,9	2,9
**3**	**scFvMEL/rGel+AlPcS_2a_**	**11**	29,9	4,9
**4**	**PDT**	**12**	26,9	2,8
**5**	**PCI of scFvMEL/rGel**	**12**	176,5	54,4

Mean time for A-375 tumors to reach the endpoint of 800 mm^3^ after various specified treatments based on Kaplan-Meier survival analysis in SPSS where all animals in the groups are included. SE represents standard error.

**Table 2:** Statistical significance analyses (p-values) to reveal differences in therapeutic effect between various treatment regimens on the A-375 tumors.

		Treatment groups			
Groups	Treatment	1	2	3	4
**1**	**untreated**				
**2**	**scFvMEL/rGel**	0,348			
**3**	**scFvMEL/rGel+AlPcS_2a_**	0,036	0,385		
**4**	**PDT**	0,132	0,493	0,796	
**5**	**PCI of scFvMEL/rGel**	<0,001	0,001	0,003	0,004

The values are based on paired log rank analyses of the time for the A-375 tumors to reach 800 mm^3^.

For the assessments of potential adverse effects, we measured the body weight of the animals, assessed skin tissue regeneration and observed the movement and behavior of the animals after the different treatment regiments. No adverse events were observed after treatment as assessed by measuring body weight (Figure 5B and C, Figure S6), or change in behavior and locomotion of the mice (data not shown). These results indicate that the normal tissue, including large blood vessels and motor neurons innervating the skeletal muscle tissue that surrounds the tumor was apparently unaffected by PCI.

Edema and erythema of the tumor tissue was observed indicating inflammation due to the photosensitization process. However this effect reversed 48–72 hours after light exposure, in accordance with reported response to PDT [31].

**Figure 6:**
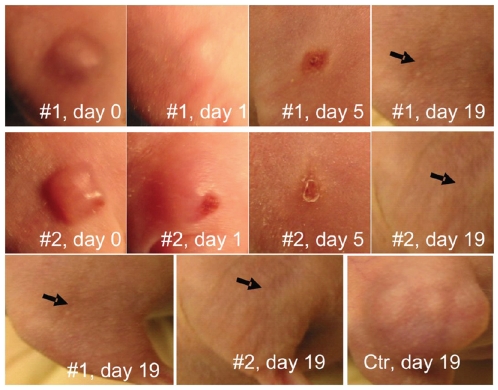
Tumor response and skin regeneration post PCI of scFvMEL/rGel. Two mice (#1 and #2) with ∼75 mm3 A-375 tumors at day 0 were further assessed at day 1, 5 and 19 post PCI. Both mice obtained CR and excellent wound repair after PCI of scFvMEL/rGel (still at day 110) as compared to a untreated control mouse (Ctr) having a tumor which reached >1000 mm3 at day 19 (∼13-fold tumor increase compared to day 0).

The skin of the mice and in particular the skin over the A-375 tumors that were treated with PCI of scFvMEL/rGel was observed over time. A non-ulcerating wound appeared on the light-exposed skin. However, the skin regenerated post PCI, leaving a small scar one week after treatment, which disappeared two weeks later (Figure 6).

## Discussion

The use of molecular cancer therapeutics is often limited by life-threatening systemic side effects due to high drug dosage and non-site-specific targeting [32]. In addition, endo-lysosomal sequestration and degradation of anti-cancer drugs is often a major obstacle for successful therapeutical outcome. Hence, there is a need for targeting drug delivery systems (DDS) that enhance drug release from endocytic drug entrapment [33]. Our labs have independently developed two different DDS for the specific targeting of cancer, the use of recombinant fusion toxins [19], [34] and photochemical internalization (PCI) for the cytosolic delivery of endo-lysosomal sequestered therapeutics [2], [4]. The overall aim of this study was to explore the concept of merging our two DDS modalities by applying PCI to cells or tumor-bearing mice treated with the fusion toxin scFvMEL/rGel. scFvMEL/rGel targets the progenitor marker HMW-MAA/NG2/MPG/gp-240, which is expressed in several types of cancers and is associated with tumor growth, metastasis and chemotherapeutic resistance [9]. In addition, we wanted to assess the potential of the present DDS to minimize possible adverse effects after treatment.

Here we report that PCI strongly enhance the cytotoxicity of scFvMEL/rGel in several gp-240 expressing cancer cell lines; A-375, MA11 and U87 in contrast to the gp-240-negative T24 cells. These findings together with the data showing that PCI of the targeting toxin is by far much more cytotoxic than the PCI of non-conjugated toxin demonstrates that the high specificity and efficacy of PCI of scFvMEL/rGel.

In the present study it has for the first time been shown that PCI is an efficient technology for enhancing the therapeutic efficacy of a systemically delivered targeting therapeutics. Using the A-375 xenograft as model, potent anti-tumor effects were obtained after PCI of scFvMEL/rGel at concentrations considerably below a maximal tolerable dose compared to a previous study where the fusion toxin alone was administered by multiple injections [19]. Improved treatment specificity, i.e. increased therapeutic efficacy and reduced side effects, is the major advantage of the DDS presented in this study. Previously, we have demonstrated *in vivo* PCI of non-conjugated gelonin after direct intratumoral injection of the protein toxin at relatively high concentrations as a proof-of-concept [3], [26], [29]. In addition, PCI mediated delivery of other non-targeting therapeutics has been demonstrated *in vivo*, i.e. bleomycin, p53-based gene therapeutics and siRNA [30], [35]–[37].The DDS system presented here may therefore reduce the dosage of anti-cancer therapeutics that is needed to achieve a satisfactory outcome.

Cancer therapy using toxin conjugates usually requires fractionated treatment, which may lead to the development of neutralizing antibodies and often inflammatory adverse effects [38]. Another obstacle in using macromolecular-based therapeutics is the slow penetration of the drug throughout solid tumors due to sterical hindrance in the extracellular matrix and elevated interstitial fluid pressure resulting in inhomogeneous distribution and low intracellular levels of the drug [39]. The PCI-technology as demonstrated here is based on low-dose single injections of the two drugs needed, i.e. injection of the photosensitizer and the targeting toxin conjugate, prior to the light activation. The enhanced anti-tumor effects and lack of adverse systemic events demonstrated in this study indicates that PCI of targeting fusion toxins provide significant improvement in anti-tumor specificity and activity, while at the same time sparing the normal tissue.

On the basis of the data presented in this study, we suggest that PCI provides a triple tumor-targeting DDS due to; 1) The targeting ability of the antibody fragment of the fusion toxin having selectivity against HMW-MAA/NG2/MPG/gp-240 expressing cancer cells; 2) A preferential accumulation and retention in malignant tissues of the photosensitizers [40], which is non-toxic in the dark and become activated only in the presence of light; 3) Confined light exposure of the tumor area with wavelengths matching the absorption of light by the PS. Indeed, the specificity of the PCI technology was confirmed by demonstrating no weight loss of animals or other side effects that were subjected to PCI of scFvMEL/rGel.

A general limitation of the PCI technology lays in the restriction of the penetration depth of the light in the tissue and the fact that this is a locoregional treatment. However, this problem may be solved by the use of optical fibers that can be inserted into the tumor tissue, as already performed during interstitial PDT [41]. Moreover, promising systemic effects reported recently by independent laboratories show that the PDT of cancer in different animal models induce an activation of systemic long-term memory T-cells and thereby have tumor-vaccinating effects [42]. Recently, this was clinically supported where untreated distant tumors regressed after local PDT of a patient with recurrent angiosarcoma [43]. The disseminated tumor cells and micro-metastasis may also be controlled by the targeting toxin. The most common side effect of PDT is photosensitivity of the skin, and hence we anticipate that this could also be the major side effect of the treatment presented in this work.

There is insufficient evidence to support the use of clinical PDT for the treatment of amelanotic melanoma (AM) in situ [44]. Interestingly, among melanomas with stem cell properties, the amelanotic cells are thought to be less differentiated and more aggressive than the melanotic cells [45]. Thus, based on the promising *in vivo* data obtained in this report, PCI of scFvMEL/rGel should be further explored as a potential treatment for HMW-MAA/NG2/MPG/gp-240 expressing cancers. A phase I clinical trial based on PCI of bleomycin has recently been approved for several indications including AM. PCI of scFvMEL/rGel could be considered as an interesting follow up for treatment of AM.

In conclusion, the present study demonstrates for the first time the tumor-targeting potential of PCI as a novel modality which may be deployed in combination with other targeted therapeutic agents. PCI of fusion toxins is a promising and innovative non-invasive, in situ treatment of cancer. Lower drug doses used to achieve anti-cancer effects made possible by the present PCI technology might lead to significant reduction of drug or treatment-induced adverse events in humans. These data appear to warrant further investigations toward future clinical applications of the PCI drug delivery system.

## Materials and Methods

### Cell lines and cell culture conditions

All cell lines were of human origin and the following were purchased from ATCC (American Type Culture Collection, Manassas VA): A-375, non-pigmented malignant skin melanoma (CRL-1619); transitional cell carcinoma of the bladder T24 (HTB-4) and U87MG, glioblastoma (HTB-14). In addition, GFP-expressing A-375 cells (A375-GFP) were established as described [20]. The lobular breast carcinoma cell line MA11 [46], was provided by Dr. Geir Olav Hjortland, Dept. of Tumor Biology, Norwegian Radium Hospital. Cells were tested as mycoplasma free.

### Chemicals

Photosensitizers Aluminum phthalocyanine with two sulfonate groups on adjacent phenyl rings (AlPcS_2a_) (Frontier Scientific, Salt Lake City, Utah) and meso-tetraphenylporphine with two sulfonate groups on adjacent phenyl rings (TPPS_2a_) (PCI Biotech ASA, Oslo, Norway) was prepared and stored as earlier described [47], [48]. Hoechst 33342 was obtained from Sigma (St. Louis, MO).

### Production of recombinant fusion toxin

A single-chain anti-HMW-MAA/NG2/MPG/gp-240 antibody (scFvMEL) was fused to recombinant gelonin (rGel), expressed in bacterial cultures, purified to homogeneity and characterized for biological activity as previously described [19].

### Fluorescence microscopy

Phase contrast and fluorescence microscopy of cells incubated with different dyes were performed by means of a Zeiss Axioplan epi-fluorescence and phase contrast microscope (Zeiss, Oberkochen, Germany) as described [47], [48]. Immunocytochemical detection of rGel by the DakoCytomation system in A-375 cells was performed by using an Olympus AX70 microscope.

### Light sources

*In vitro* light exposures of tumor cells were performed by using LumiSourceTM lamp (PCI Biotech, Oslo, Norway) emitting either blue (11.7 mW/cm^2^) or red light (1.5 mW/cm^2^) as previously described [47], [49]. The *in vivo* illumination was performed with a 300 mW diode laser, Ceralas I 670 (CeramOptec GmbH, Bonn, Germany) emitting 670 nm light.

### *In vitro* drug delivery by PCI

Cells were seeded in 96-well plates (Nunc, Roskilde, Denmark) at 3500 (T24), 4000 (A-375/A375-GFP, MA11) or 8000 (U87MG) cells/cm^2^ and allowed to attach to the substratum at 37°C, either for 6 hours or over night. scFvMEL/rGel was then co-incubated with TPPS_2a_ or AlPcS_2a_ for 18 hours. This was followed by a three-time drug-free medium wash of the cells and a further 4 hour drug-free medium chase. Subsequently, the cells incubated with TPPS_2a_ or AlPcS_2a_ were subjected to light exposure by using the blue or the red light LumiSource respectively.

### Cytotoxic assays

Cytotoxicity was evaluated using the MTT assay 48 h post PCI, and when possible, confirmed by clonal cell survival as previously described [47]. For MTT assay, agents have known to interfere with the assay [50], however the agents used in this work do not have any absorbance at 570 nm. In addition, PCI of scFvMEL/rGel was qualitatively assessed by fluorescence microscopy of the A375-GFP cells post treatment, since cellular leakage of GFP due to plasma membrane damage has been established as an method for assessment of cell death [22], [23].

### Immunocytochemistry (ICC)

A-375 cells (10000 cell/well) were seeded out in 16-well chamber slides from Lab-Tek (Nunc) and treated with PCI as described above. Cell plasmamembranes were stripped with 100 µl 0.1 M Glycine (pH 2.5) in 0.5 M NaCl for 5 minutes at 37°C, neutralized with 100 µl 0.5 M Tris-HCl (pH 7.4) for 5 minutes at 37°C. The cells were subsequently fixed with 4% buffered formalin at room temperature and stained using the Dako EnVision+ System HRP-DAB (K4011, DakoCytomation, Carpinteria, CA). Cells were treated with 0.03% hydrogen peroxide for 5 min and then incubated with a polyclonal rabbit-anti-gelonin IgG (1∶400, 0.5 mg/ml, National Institute of Health, Oslo, Norway) for 30 min at room temperature. The cells were then incubated with Peroxidase-labeled polymer conjugated to goat anti-rabbit IgG for 30 min and stained for 10 min with 393-diaminobenzidine tetrahydrochloride, counterstained with hematoxylin, dehydrated, and mounted in Diatex (Sigma). The antibody has previously been validated for detection of native gelonin in tumors [29].

### Animal model

Female BALB/c (nu/nu) nude mice were bred at the Animal Department of the Norwegian Radium Hospital and maintained under specific pathogen-free conditions, with food and water supplied ad libitum. Housing and all procedures involving animals were performed according to protocols approved by the institutional animal care and use committee, in compliance with the Norwegian Animal Research Authority's guidelines. The mice were on average 20 to 25 g (5–8 weeks old) at the start of the experiments.

### *In vivo* delivery of scFvMEL/rGel by PCI

Cultured subconfluent A-375 cells were prepared for s.c. injection as previously described [19]. Treatment commenced at an A-375 tumor volume of 50–100 mm3. Forty-eight hours prior to PCI-treatment, the mice were subjected to i.p. injection of 5 mg/kg bw of AlPcS_2a_ followed the next day by i.v. administration of 2 mg/kg bw of scFvMEL/rGel 24 h prior to 670 nm laser exposure at an irradiance of 100 mW/cm^2^ and the total dose of 20 J/cm^2^. To avoid undesired light exposure of normal tissue during the light exposure, the whole body was covered with aluminum foil, except for the skin over the tumor area and a few millimeter rim of normal skin surrounding the tumor. The tumor volume was calculated using the following formula:
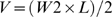
according to the US National Cancer Institute protocols where W is the with and L the length of the tumors measured 2 or 3 times per week with a digital caliper. The animals were euthanized by cervical dislocation if the tumor volume exceeded 1000 mm^3^.

### Statistical analysis

*In vitro* and *in vivo* data were expressed as mean+/−SD or +/−SE, respectively. All *in vitro* experiments are based on triplicates and reproduced at least twice. Representative results are depicted. Differences between groups were *in vitro* estimated by Student's t-test. All differences were deemed significant at a level of p≤0.05. Analyses were performed by the Sigma Plot Software Package.

The tumor growth data were subjected to survival analysis, using the day when the tumor volume supersedes the volume V_crit_ = 800 mm3 defined as the failure time, and the duration of the experiment as the censoring time, if the tumor does not obtain this volume. Statistical differences in treatment response were evaluated by pair wise log-rank analyses in SPSS 15.0.

Because both scFvMEL/rGel and PDT induced a delay in tumor growth, documentation of synergism requires more detailed analyses, as previously described [30]. The documentation of synergy is based on a log-logistic survival function. Synergy or antagonism is regarded as statistically significant when the additional interaction variable, *β_3_*, is significantly different (p<0.05) from the sum of the individual survival variables, *β_12_* = *β_1_*+*β_2_*. The synergistic effect can be described by R = *β_3_*/(*β_1_*+*β_2_*), where R = 0 describes additivity and R<0 and R>0 respectively, when significant, describes antagonism and synergism. As an example R = 0.5 describes a synergy effect of the same order of magnitude as 50% of the two treatments combined.

## Supporting Information

Figure S1 TPPS2a-PCI of increasing doses of toxins. Light-dose and toxin concentration-dependent reduction of cell viability post PCI of scFvMEL/rGel in A-375 using TPPS2a as photosensitizer. A, PCI of 100 nM native (n), recombinant (r) gelonin (rGel) or scFvMEL/rGel after 18 hours co-incubation with 0.2 mg/ml TPPS2a and 4 hours chase in drug-free medium. B, Experiments with triplicates were reproduced at least twice. MTT assay was performed 48 hours post light exposure. Bars, SD.
(0.05 MB PPT)

Figure S2 Cytotoxic response 24 hours post light exposure. TPPS2a-PCI of 100 nM scFvMEL/rGel or rGel/nGel in A-375 cells (PS-concentration: 0.2 mg/ml). Cells were treated as described in the Materials and Methods, however the MTT assay was performed 24 hours and not 48 hours, post light exposure. Bars, SD.
(0.04 MB PPT)

Figure S3 EGFP signals post PDT and PCI 24 and 48 hours after light exposure. The reduction of EGFP signals after PCI of scFvMEL/rGel is much more pronounced 48 hours than at 24 hours post light exposure, while no differences in green fluorescence between 24 and 48 hours post PDT was detected. Cells treated with PDT, as shown in the first and third row are either dead, dying or alive, while cells treated with PCI are most truly dying or dead. Representative fluorescence micrographs are shown for each time point. Treatment conditions were as described in Figure 2.
(9.10 MB PPT)

Figure S4 Cytotoxic response when scFvMEL-rGel was administered after the photochemical treatment to A-375 cells. The cells were incubated with 0.2 mg/ml TPPS2a for 18 hours, washed twice and chased with drug-free medium for 4 hours prior to light exposure. Immediately after light exposure the cells were treated with scFvMEL/rGel or rGel (both 100 nM) for 18 hours before medium was changed with drug-free medium. MTT activity was measured 48 hours post light exposure. Bars, SD.
(0.04 MB PPT)

Figure S5 Detection of selective binding of scFvMEL/rGel to MA11 cells. A, Cells were incubated with 80 nM scFvMEL/rGel on ice for 30 min. Cells were then washed twice with ice cold medium and further incubated with a rabbit anti-gelonin antibody (1∶50 dilution) for 30 min on ice. Subsequently, the cells were washed twice with ice cold medium and further incubated with a secondary Alexa488 labeled goat anti-rabbit antibody (1∶100) and incubated on ice for 30 min. The cells were then washed twice with ice cold PBS (w.Ca2+) and subjected to fluorescence microscopy. B, Control binding. Cells that were not incubated with scFvMEL/rGel bit received the same antibody treatment as described above. The same fluorescence intensity range was set for all micrographs.
(1.02 MB PPT)

Figure S6 Assessment of the weights of the animals. The body weights of the mice were monitored twice weekly. Mice were treated as indicated in the figure and otherwise described in Figure 5.
(0.05 MB PPT)
