# Supplementary figures and images for: Multi-Modality Therapeutics with Potent Anti-Tumor Effects: Photochemical Internalization Enhances Delivery of the Fusion Toxin scFvMEL/rGel
Source: PLoS One. 2009 Aug 19;4(8):e6691. doi: 10.1371/journal.pone.0006691 (PMC2723936; doi:10.1371/journal.pone.0006691)

## Slide 1
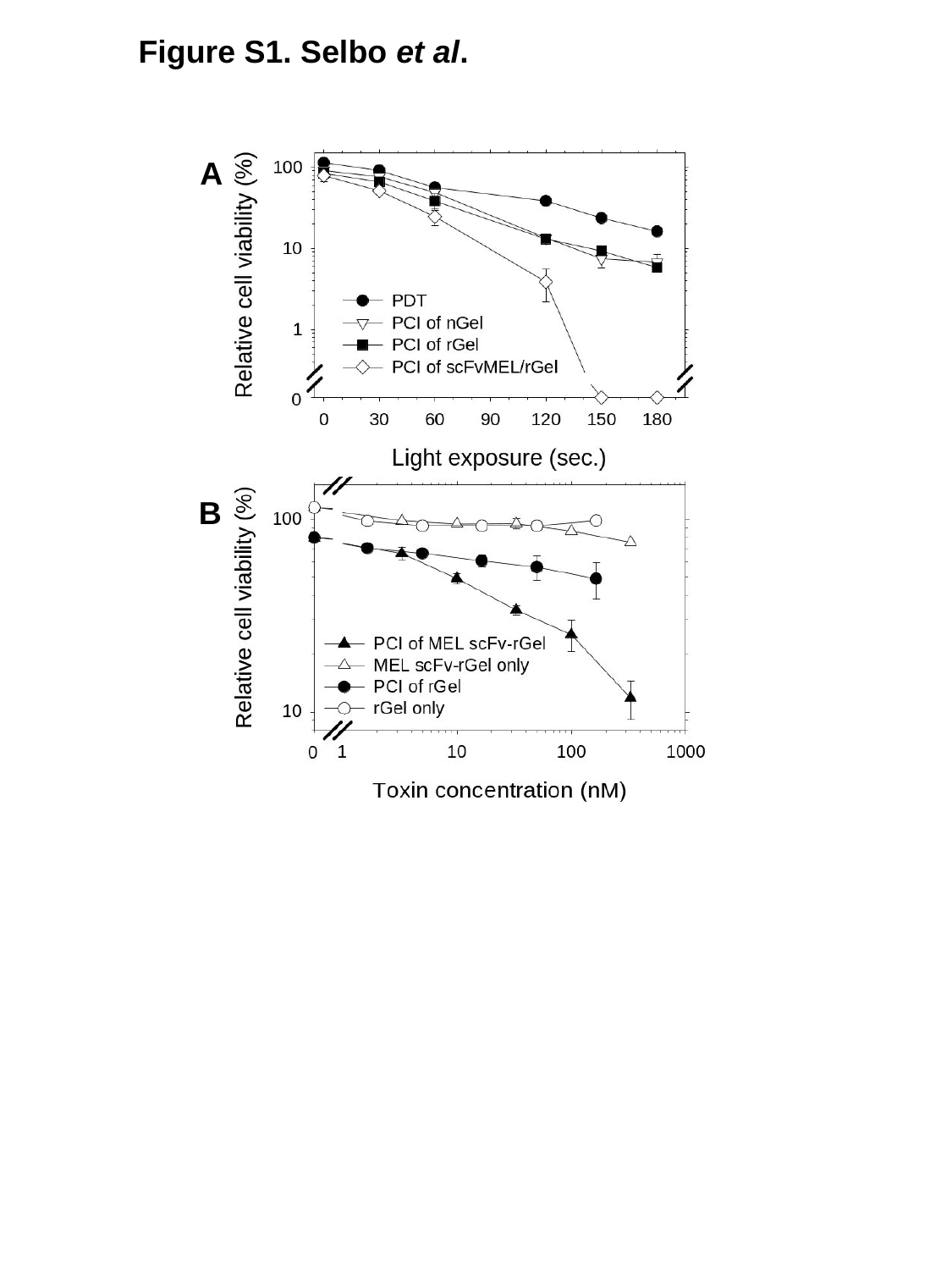

Figure S1. Selbo et al.
A
B

Supplement: Figure S1 — TPPS2a-PCI of increasing doses of toxins. Light-dose and toxin concentration-dependent reduction of cell viability post PCI of scFvMEL/rGel in A-375 using TPPS2a as photosensitizer. A, PCI of 100 nM native (n), recombinant (r) gelonin (rGel) or scFvMEL/rGel after 18 hours co-incubation with 0.2 mg/ml TPPS2a and 4 hours chase in drug-free medium. B, Experiments with triplicates were reproduced at least twice. MTT assay was performed 48 hours post light exposure. Bars, SD. (0.05 MB PPT) [file pone.0006691.s001.ppt]

## Slide 1
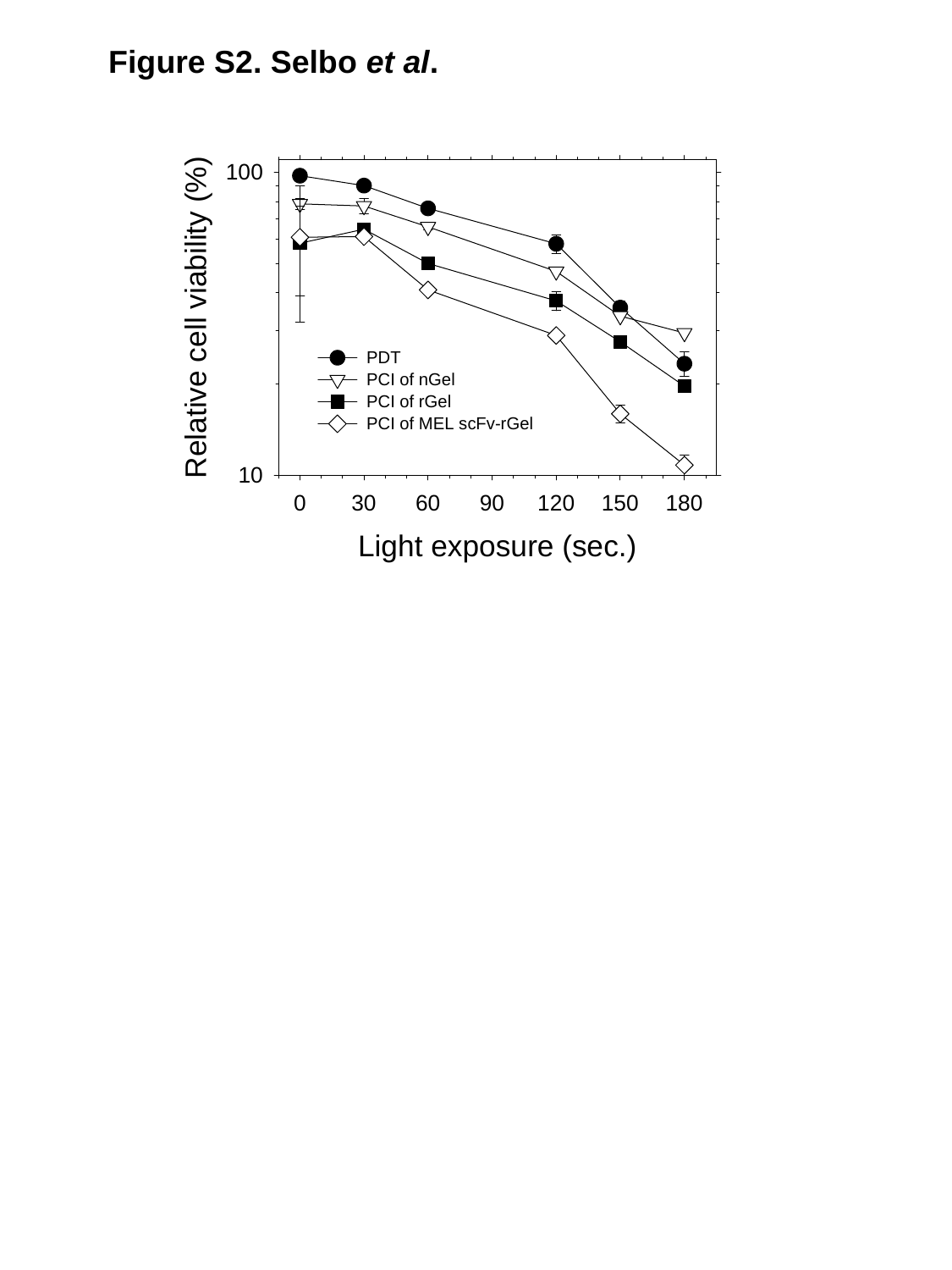

Figure S2. Selbo et al.

Supplement: Figure S2 — Cytotoxic response 24 hours post light exposure. TPPS2a-PCI of 100 nM scFvMEL/rGel or rGel/nGel in A-375 cells (PS-concentration: 0.2 mg/ml). Cells were treated as described in the Materials and Methods, however the MTT assay was performed 24 hours and not 48 hours, post light exposure. Bars, SD. (0.04 MB PPT) [file pone.0006691.s002.ppt]

## Slide 1
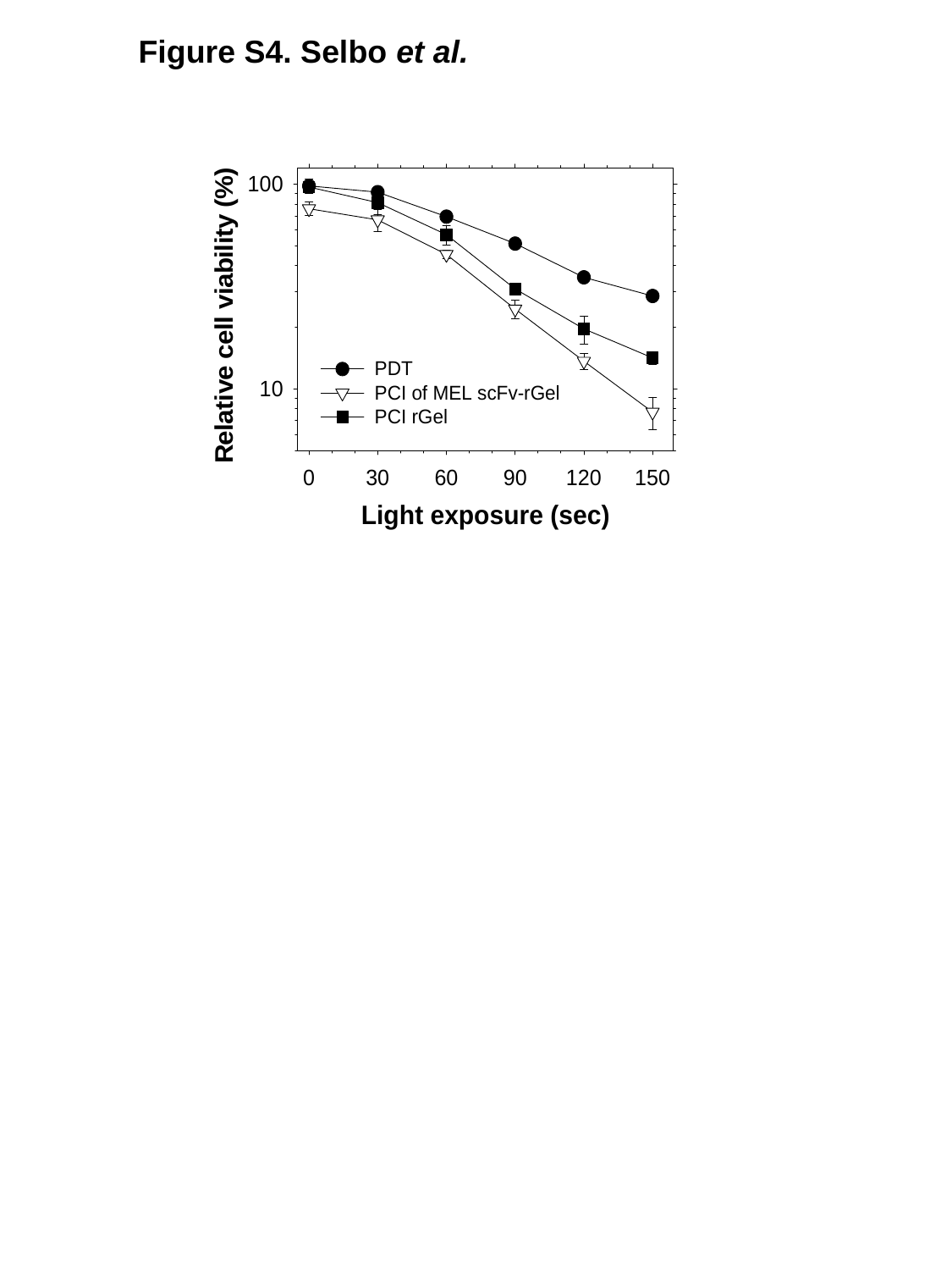

Figure S4. Selbo et al.

Supplement: Figure S4 — Cytotoxic response when scFvMEL-rGel was administered after the photochemical treatment to A-375 cells. The cells were incubated with 0.2 mg/ml TPPS2a for 18 hours, washed twice and chased with drug-free medium for 4 hours prior to light exposure. Immediately after light exposure the cells were treated with scFvMEL/rGel or rGel (both 100 nM) for 18 hours before medium was changed with drug-free medium. MTT activity was measured 48 hours post light exposure. Bars, SD. (0.04 MB PPT) [file pone.0006691.s004.ppt]

## Slide 1
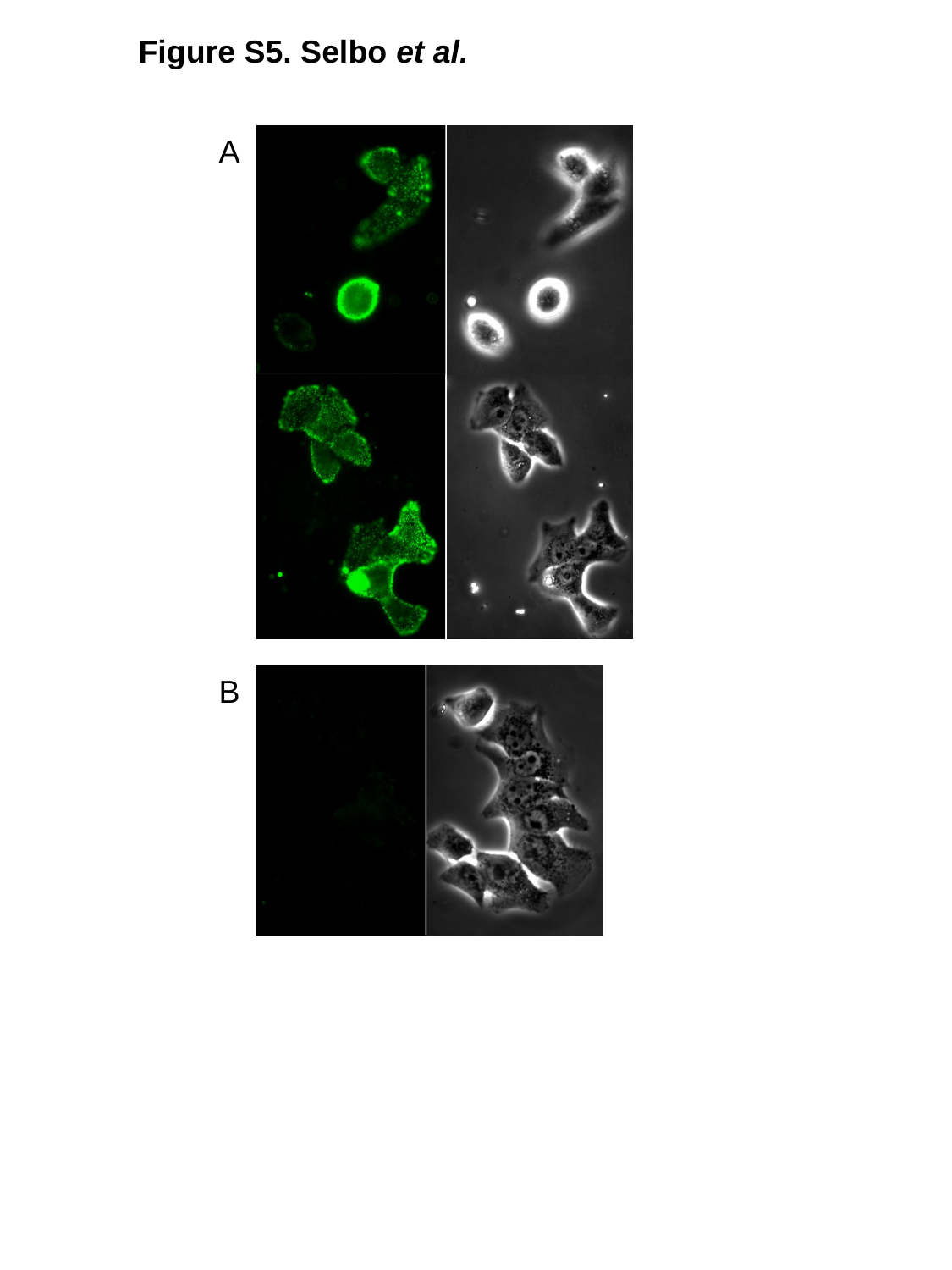

Figure S5. Selbo et al.
A
B

Supplement: Figure S5 — Detection of selective binding of scFvMEL/rGel to MA11 cells. A, Cells were incubated with 80 nM scFvMEL/rGel on ice for 30 min. Cells were then washed twice with ice cold medium and further incubated with a rabbit anti-gelonin antibody (1∶50 dilution) for 30 min on ice. Subsequently, the cells were washed twice with ice cold medium and further incubated with a secondary Alexa488 labeled goat anti-rabbit antibody (1∶100) and incubated on ice for 30 min. The cells were then washed twice with ice cold PBS (w.Ca2+) and subjected to fluorescence microscopy. B, Control binding. Cells that were not incubated with scFvMEL/rGel bit received the same antibody treatment as described above. The same fluorescence intensity range was set for all micrographs. (1.02 MB PPT) [file pone.0006691.s005.ppt]

## Slide 1
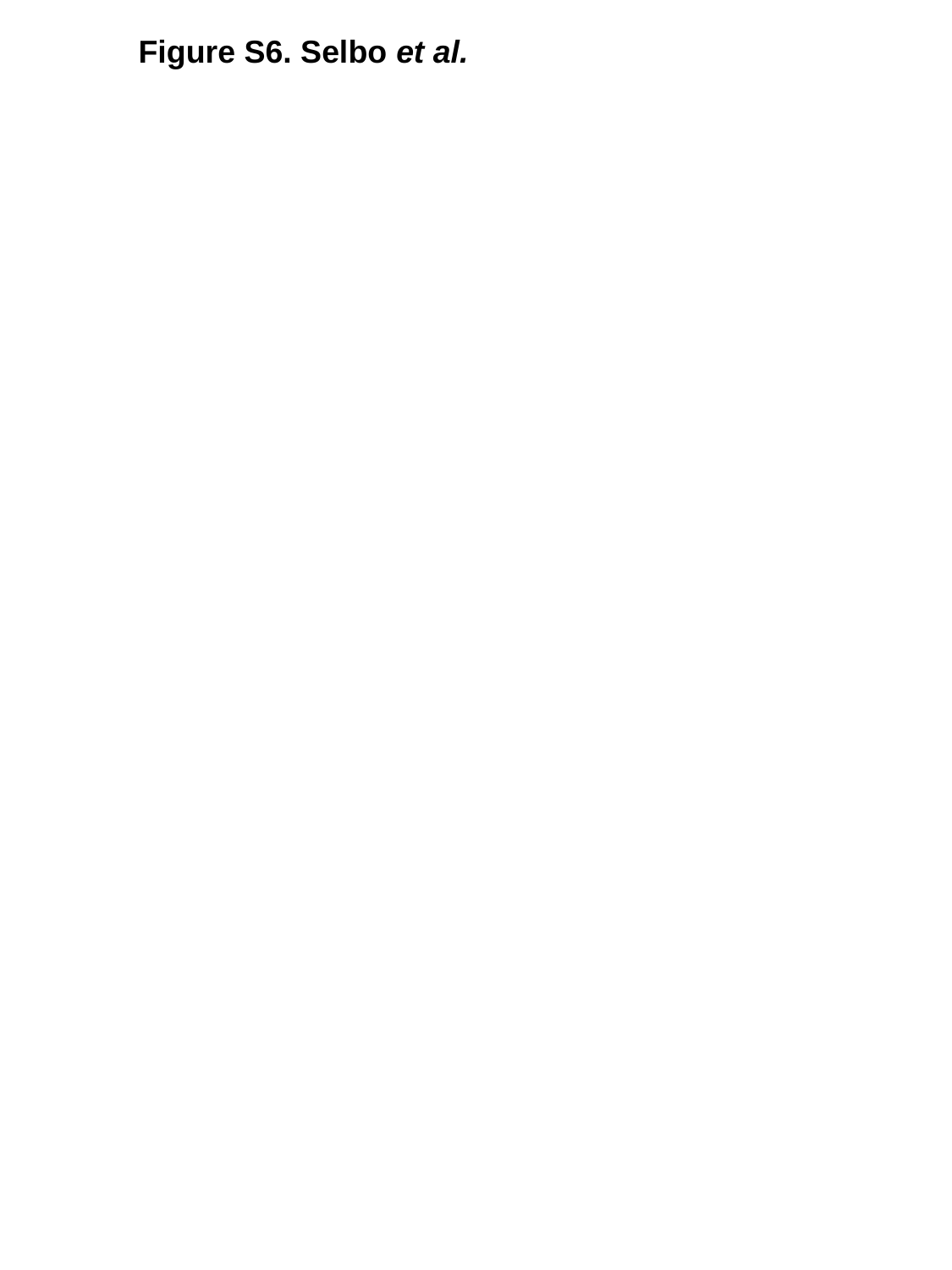

Figure S6. Selbo et al.

Supplement: Figure S6 — Assessment of the weights of the animals. The body weights of the mice were monitored twice weekly. Mice were treated as indicated in the figure and otherwise described in Figure 5. (0.05 MB PPT) [file pone.0006691.s006.ppt]
