# Supplementary material for: Multi-Modality Therapeutics with Potent Anti-Tumor Effects: Photochemical Internalization Enhances Delivery of the Fusion Toxin scFvMEL/rGel
Source: PLoS One. 2009 Aug 19;4(8):e6691. doi: 10.1371/journal.pone.0006691 (PMC2723936; doi:10.1371/journal.pone.0006691)
Supplement: Figure S3 — EGFP signals post PDT and PCI 24 and 48 hours after light exposure. The reduction of EGFP signals after PCI of scFvMEL/rGel is much more pronounced 48 hours than at 24 hours post light exposure, while no differences in green fluorescence between 24 and 48 hours post PDT was detected. Cells treated with PDT, as shown in the first and third row are either dead, dying or alive, while cells treated with PCI are most truly dying or dead. Representative fluorescence micrographs are shown for each time point. Treatment conditions were as described in Figure 2. (9.10 MB PPT) [file pone.0006691.s003.ppt]

## Slide 1
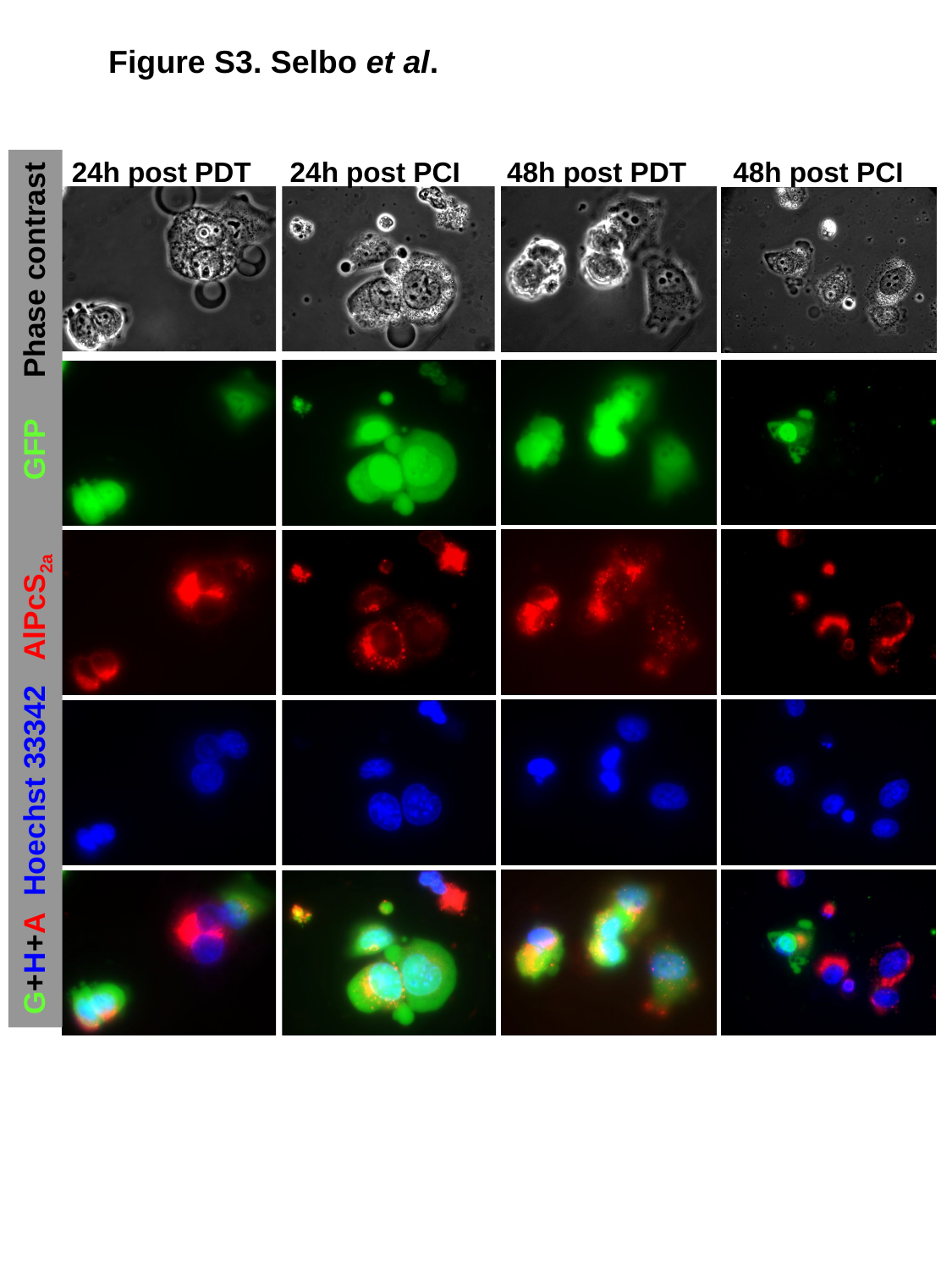

Figure S3. Selbo et al.
24h post PDT 24h post PCI 48h post PDT 48h post PCI
G+H+A Hoechst 33342 AlPcS2a GFP Phase contrast
